# Achieving zero new HIV infection, unsafe sexual practices of out of school border youths

**DOI:** 10.1186/1742-4690-9-S1-P116

**Published:** 2012-05-25

**Authors:** AO Sekoni, AT Onajole

**Affiliations:** 1 College of Medicine, University of Lagos, Lagos, Nigeria

## Background

Young people aged 15 to 24 account for more than 50 percent of all new HIV infections worldwide, majority are those who engage in unsafe sex (unprotected casual sex and multiple sex partners), unsafe injection drug use, exposure to contaminated blood and blood products or unsterilized skin piercing procedures. Border towns have an admixture of vulnerable population including uniform personnel, out of school youths, traders, drivers, commercial sex workers and migrants as well as risky sites such as bars, hotels, brothels and truck parking areas.

## Methodology

This cross sectional study was carried out among border youths to assess knowledge and practices of safer sex and use of HCT services. One in two systematic market stall sampling was used in the border market between Nigeria and Republic of Benin to select participants for the study, in each of the selected stalls all the youths 15 to 24 years were interviewed using a validated structured questionnaire until sampling size of 120 was achieved.

## Results

The mean age was 19years, majority were female (62%) & single (73%). Half of them had at least secondary school education, 22% had no formal education, 93% were Nigerians, 25% live on their own, while 36% were financially responsible for themselves. Two third (68%) did not know what safer sex means although 43% have had sexuality education, mean age at sexual debut was 16yrs, 64% have had sex while 63% were still currently sexually active. The main reason for having sex was to have fun (56%), more than a quarter engage in multiple sexual partnerships, 61% use condoms, among which only 15% were consistent users and 18% have accessed HCT services. Those who consume alcohol were more likely to be sexually active as well as have multiple sexual partners.

## Conclusion

The out of school border youths in this study engage in risky sexual behavior that can put them at risk of HIV infection, uptake of HCT (a prevention strategy) is poor.

